# Human amniotic membrane products for patients with diabetic foot ulcers. do they help? a systematic review and meta-analysis

**DOI:** 10.1186/s13047-022-00575-y

**Published:** 2022-09-14

**Authors:** Yasmine Adel Mohammed, Hossam Khaled Farouk, Mohamed Ibrahim Gbreel, Abdelrahman Mahmoud Ali, Ali Ashraf Salah, Anas Zakarya Nourelden, Mohamed Mahmoud Abd-El Gawad

**Affiliations:** 1 Faculty of Medicine, Assiut University, Assiut, 71631 Assiut governorate Egypt; 2 International Medical Research Association (IMedRA), Cairo, Egypt; 3 Faculty of Medicine, Al-Fayoum University, Fayoum, Egypt; 4 Faculty of Medicine, October 6 University, Giza, Egypt; 5 Faculty of Medicine, Minia University, Minia, Egypt; 6 Faculty of Medicine, Al-Azhar University, Cairo, Egypt

**Keywords:** Diabetic foot ulcer, Human amnion membrane, Amniotic allograft, Grafix, AmnioBand, EpiFix

## Abstract

**Background:**

Diabetic foot ulcer (DFU) is one of the most serious diabetic complications. DFU is an open wound that usually occurs in the foot sole due to poor blood glucose control, peripheral neuropathy, and poor circulation. The human amniotic allograft membrane is a biological wound dressing derived from the amniotic membrane. It contains amino acids, nutrients, cytokines, and growth factors that make the growth process easier.

**Objective:**

To compare dehydrated human amnion and chorion allograft (DHACA) plus the standard of wound care (SOC) with the SOC alone.

**Methods:**

We searched for randomized clinical trials (RCTs) on PubMed, Scopus, Cochrane, and Web of Science till April 2021 using relevant keywords. All search results were screened for eligibility. We extracted the data from the included trials and pooled them as mean difference (MD) or risk ratio (RR) with the 95% confidence interval (CI) using Review Manager software (ver. 5.4).

**Results:**

The pooled effect estimate from 11 RCTs showed that DHACA was superior to SOC regarding the complete wound healing in both 6th and 12th week (RR = 3.78; 95% CI: [2.51, 5.70]; *P* < 0.00001) and (RR = 2.00; 95% CI: [1.67, 2.39], *P* < 0.00001 respectively). Also, the analysis favored the DHACA regarding the mean time to heal in the 12th-week (MD = -12.07, 95%CI: [-19.23, -4.91], *P* = 0.001). The wound size reduction was better with DHACA (MD = 1.18, 95%CI: [-0,10, 2.26], *P* = 0.03).

**Conclusion:**

Using DHACA with SOC is safer and more effective than using SOC alone for DFU patients.

**Supplementary Information:**

The online version contains supplementary material available at 10.1186/s13047-022-00575-y.

## Introduction

Diabetes mellitus (DM) is a worldwide epidemic disease. In 2019, the global diabetes prevalence was projected to be 9.3% (463 million people). The prevalence is estimated to rise to 10.2% (578 million) by 2030 and 10.9% (700 million) by 2045 [1]. Diabetic foot ulcer (DFU) is one of the most serious and common complications of diabetes that itself can be complicated by wound infection, gangrene, and unfortunate amputation. Amputation can comprise a huge burden on the patients' quality of life and the health systems' economy [2]. The global prevalence of DFU is 6.3%, affecting males more than females, and patients with type 2 DM more than type 1 [3]. Diabetic foot ulcer is primarily caused by hyperglycemia that results from endothelial dysfunction, leading to vascular insufficiency and nerve injury [4, 5].

The current DFU standard of care (SOC) involves four principles; pressure relief, debridement, infection management, and revascularization when indicated. Preventative measures such as adequate glycemic control, periodic foot inspection, as well as patient and family education are always recommended [6, 7]. Sometimes the SOC is not enough for the management of DFUs, therefore, new trends have emerged to address this problem. These include negative pressure wound therapy, hyperbaric oxygen therapy, bioengineered skin substitutes, and shockwave therapy, among several other measures. These novel therapies have shown significant DFU clinical improvement in different subsets of DFU. However, much of the literature came from smaller trials with inconsistent patient selection and outcomes measurement, making it difficult to assess the exact clinical benefit of these treatments [7].

Although we associate regenerative medicine with the recent decades, amnion has been used in the medical field for over a century. The first known usage for amnion was in a skin transplant, in 1910 at John Hopkins Hospital [8]. Dehydrated human amnion-chorion membranes and placenta possess marvelous features, from the pluripotent stem cells which can differentiate into all three germ layers, to the angiogenic anti-inflammatory properties coming from a wide variety and mixture of angio-modulatory cytokines, anti-bacterial peptides, and anti-inflammatory agents [9, 10]. These membranes are currently considered a new hope in regenerative medicine owing to their wide uses, low immunogenicity, and easy procurement from the placenta. As the placenta is a discarded tissue after parturition, the current controversies associated with the use of human embryonic stem cells are avoided [11].

Dehydrated human amniotic and chorionic allograft (DHACA) is easier for application and commercially available. This product can be applied directly to clean the debrided wounds where the infection has been controlled and adequate vasculature and perfusion state exist, to achieve wound healing as early as possible [12]. Many studies have shown that DHACA as a treatment for diabetic foot ulcers is more effective than standard wound care alone. For further evaluation of the efficacy and time-sensitivity of DHACAs in patients suffering from DFU, we performed this systematic review and meta-analysis study. Our study compares using DHACA plus SOC versus SOC alone.

## Material and methods

We performed a systematic review and meta-analysis for clinical trials on the use of dehydrated human amnion/chorion membrane for the treatment of DFU. We followed the Preferred Reporting Items for Systematic Reviews and Meta-Analyses (PRISMA) guidelines in reporting our study [13].

### Search strategy

We used four different databases for the literature search (PubMed, Scopus, Cochrane and Web of Science), and the search was conducted from their inception till October 2020. The following keywords were used (Diabetic foot ulcer, human amnion membrane, amniotic allograft, Grafix, AmnioBand, EpiFix), and MESH terms were used when applicable. We also did a manual screening of references in the included studies, searching for any relevant trials.

### Inclusion and exclusion criteria

English-written human-based randomized clinical trials (RCTs) were included in our study. Diabetic patients with foot ulcers were the target population. The intervention was human amnion, chorion, placental membrane, or any brand using them like Grafix, GrafixPL PRIME, AmnioBand, Stravix, biological dressing, bio implant dressing, or EpiFix. The comparator was any effective measurement like SOC. We excluded conference abstracts, books, single-armed clinical trials, animal studies, and studies on non-diabetic patients.

### Study selection

We used EndNote X8 for citation management and duplicate removal for articles identified in the searches. We selected the included studies in a two-stage screening process. In the first stage, the titles and abstracts from the electronic searches were screened independently. The second stage of full-text screening was performed to determine the final decision on studies' eligibility. The assessment of each manuscript was performed independently by at least three authors, and any disagreements about inclusion were resolved by consultation with the principal investigator of the study.

### Quality assessment

The risk of bias was assessed according to the Cochrane risk of bias tool described in the Cochrane Handbook for Systematic Reviews of Interventions 5.1.0 [14]. There are six domains in the tool: random sequence generation, allocation sequence concealment, blinding of study participants and personnel, blinding of outcome assessors, incomplete outcome data, selective outcomes reporting, and other potential sources of bias. We reported the quality of the included studies as low risk, high risk, or unclear risk of bias. In addition, we measured the publication bias through visualization of the funnel plot for any asymmetrical distribution [15].

### Data extraction

Each author – independently – extracted data from all the included trials. Data extraction was performed in an excel sheet that included three sections. Firstly, general data included the year of publication, protocol registration, definition of ulcers, groups and sample size, and intervention. Then, baseline data included age, race, gender, Body Mass Index (BMI), mean glycated hemoglobin, smoking, duration of wound, initial wound surface area in cm2, and wound location.

### Primary and secondary outcomes

The primary outcomes were the percentage of complete wound healing by the 6^th^ and 12^th^ week and the mean time to heal within the 1^st^, 6^th^, and 12^th^ weeks. The secondary outcomes included the Kaplan–Meier plot of time to heal within the 1^st^, 6^th^, and 12^th^ week, and wound size reduction. An adverse events analysis was performed, including any unfavorable outcome that occurred to patients in each group during the time of the trial like (Cellulitis, osteomyelitis and infection of the affected extremity, development of another ulcer, deep vein thrombosis, urinary tract infection and gastrointestinal bleed).

### Statistical analysis

We conducted the meta-analyses using the Review Manager (RevMan) computer program (Version 5.4. Copenhagen: The Nordic Cochrane Centre, The Cochrane Collaboration, 2014). Regarding pooling of the study outcomes, risk ratio (RR) with the 95% confidence interval (CI) were used for dichotomous variables, while the mean difference (MD) and the 95% CI were presented for continuous variables. Cochrane's *P* values and the I^2^ were tested to examine heterogeneity among the included studies. High heterogeneity existed in some analyses most likely due to clinical and methodological factors, therefore, the random effect model was adopted in these analyses. Funnel plots and the Egger regression test were conducted and measured through visualization of the funnel plot. Besides, a sensitivity analysis was performed by sequentially deleting trials to check for the stability of the primary outcomes.

## Results

### Literature search

**Fig. 1:**
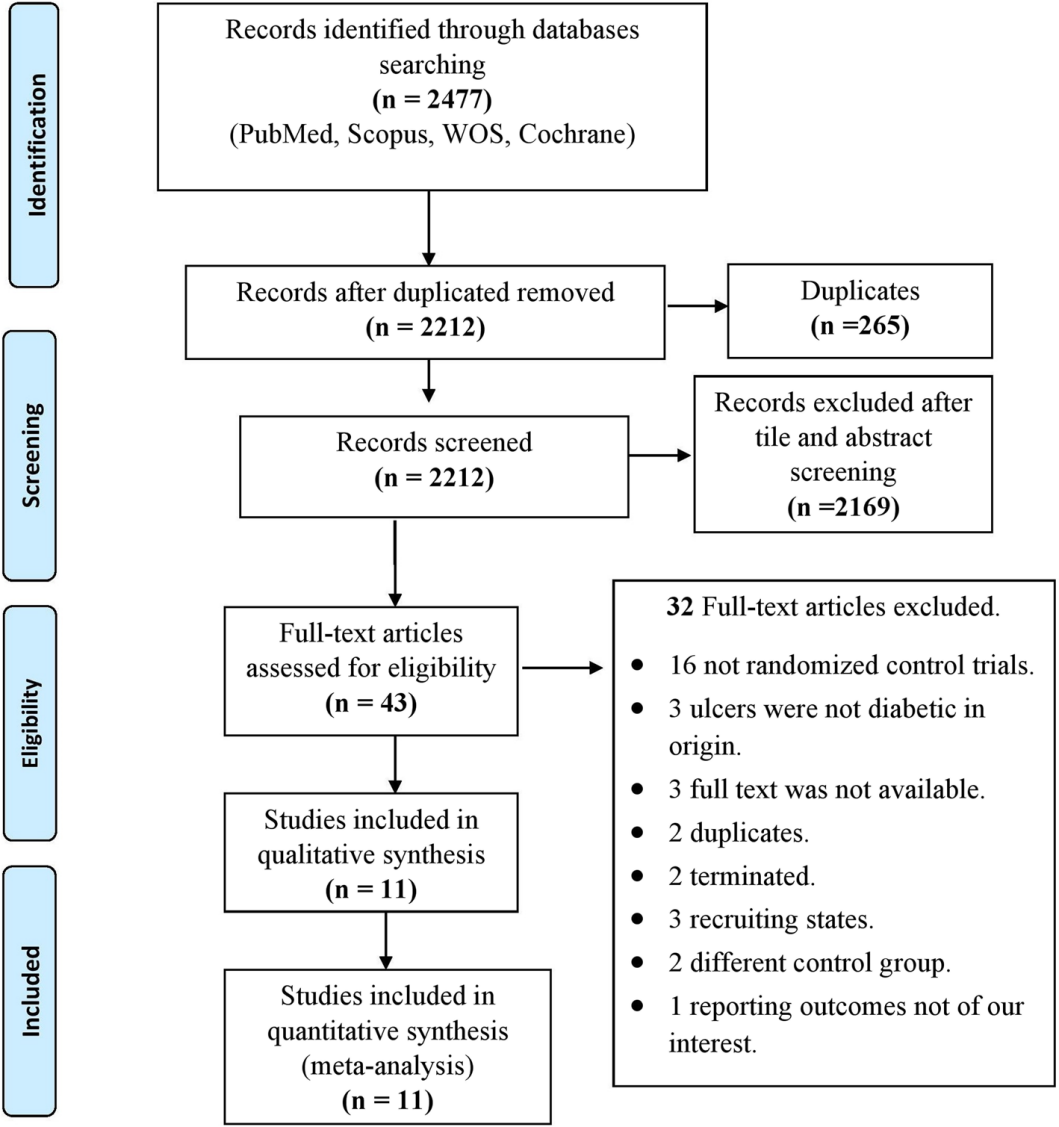
PRISMA flow chart of the literature search

The literature search revealed 2477 results, 265 of them were duplicates. Therefore, we performed a title and abstract screening for 2212 results, of which only 43 results were judged relevant. These 43 studies were eligible for full-text screening which finally resulted in including only 11 studies in our review. We excluded 16 studies that were not RCTs, three studies because ulcers were not diabetic in origin, three studies because full-texts were not published (only abstracts available), two studies that were duplicates of already existed studies, two studies that were terminated, three studies that were in recruiting state, two studies for the different control group, and one study for reporting outcomes that were not of our interest. Fig. 1

### Risk of bias

**Fig. 2:**
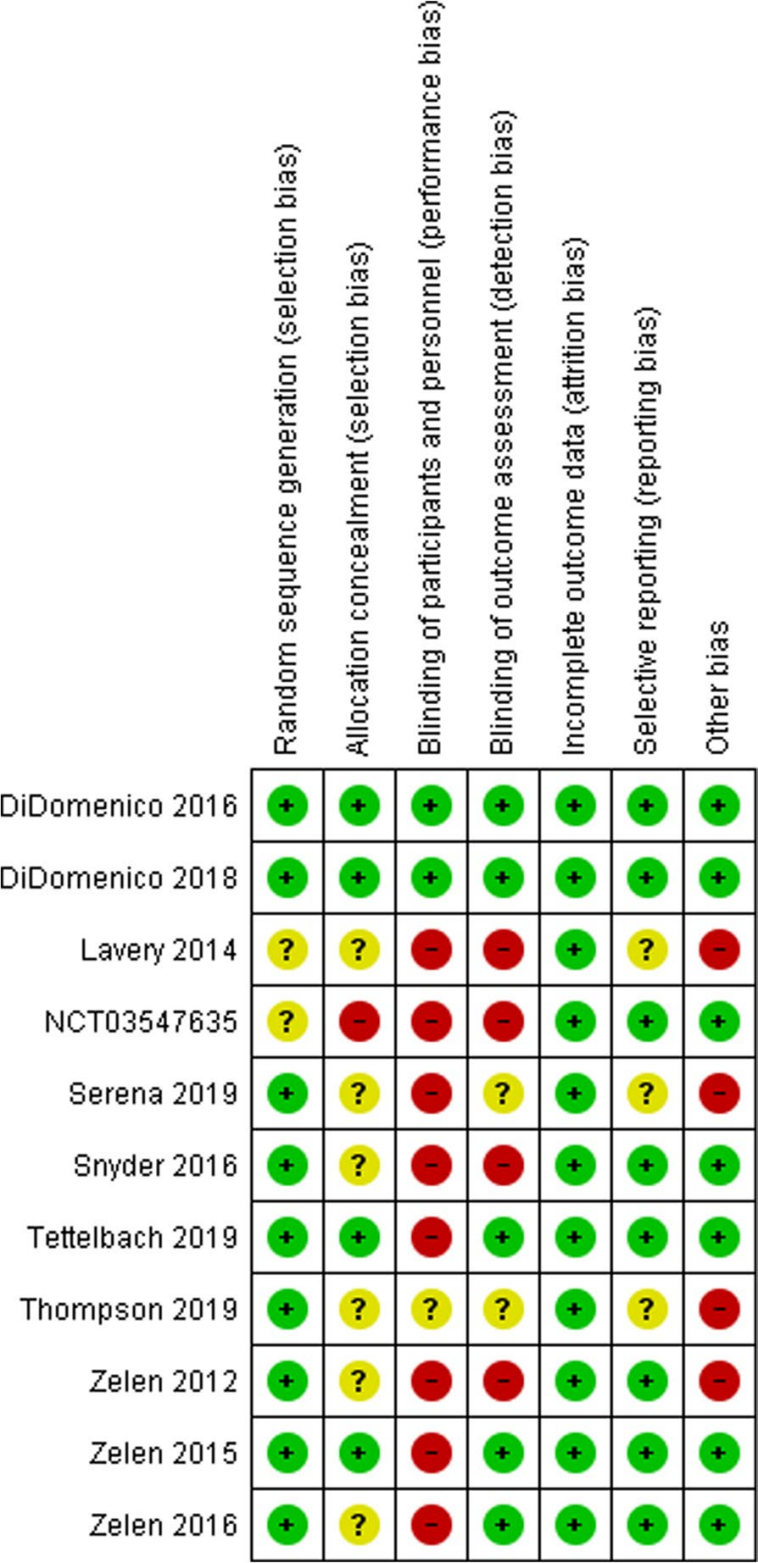
Cochrane risk of bias summary of the included studies

Overall, the included studies were of moderate quality. Regarding the selection bias, most of the studies reported appropriate randomization methods and were at low risk of bias. However, Lavery et al. [16] and NCT03547635 which did not report the method of randomization, and thus had an unclear risk of bias. Six studies [16–21] did not report the method of allocation concealment, and thus had an unclear risk of bias. However, one study NCT03547635 reported no allocation concealment, and thus was at high risk of bias. In terms of performance bias, eight studies reported an inability to blind the participants due to the nature of the intervention, however, blinding was possible in other two studies [22, 23]. Therefore, incomplete participants and personnel blinding were considered a high risk of bias. One study did not mention anything about the blinding, thus was considered unclear risk of bias [20]. No missing data were detected, as all the studies reported using the intention to treat analysis. Regarding the detection bias, four studies reported that the analysis was performed by an unblinded statistician [16, 17]. Fig. 2

**Fig. 3:**
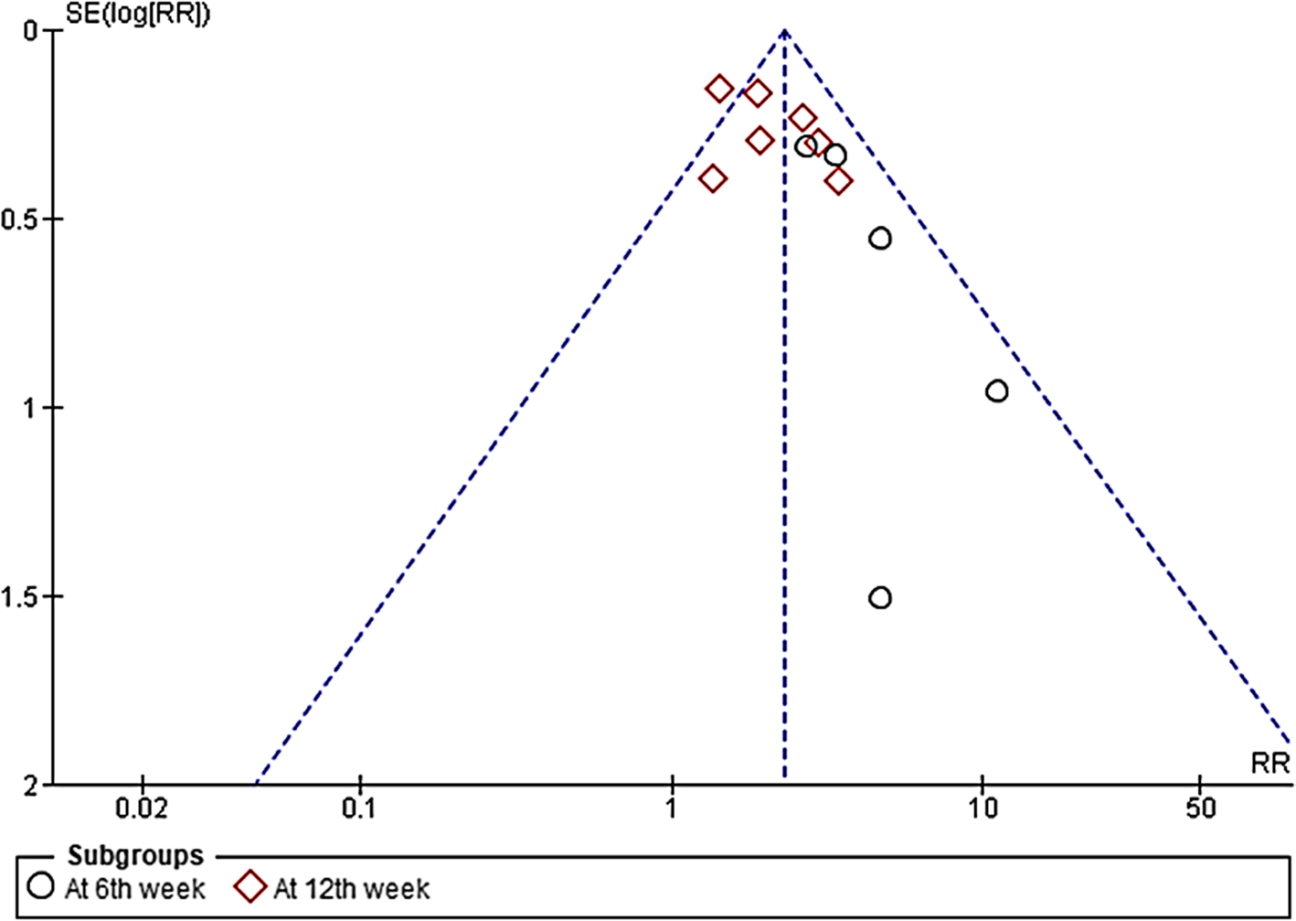
Funnel plot for publications bias assessment of the included studies in the outcome of the complete wound healing

Finally, we considered the low sample size and the absence of protocol a high risk of other bias. Therefore, four studies [16–18, 20] were considered at high risk. None of the studies' authors had a conflict of interest with any of the suppliers of the amniotic membrane products. Publication bias was measured through visualization of the funnel plot and it was visually not symmetrical. Fig*. **3*

### Studies characteristics

A total of 655 patients suffering from DFU were eligible for this review. From this total number, 328 patients underwent DHACA with SOC and 327 patients underwent SOC alone. Patients were followed up from six to 16 weeks. The majority of the trials followed the patients for 12 weeks. However, Snyder et al. [12] followed the patients for six weeks, while Serena et al. [18] followed them for 16 weeks. The summary of the eligible trials including NCT, sample size, follow-up duration, definition of ulcer, amniotic membrane products, and type of the applied intervention are presented in Table*1**.* Baseline characteristics of the studies’ enrolled patients including study arms, the number of patients in each arm, age, gender, BMI, glycated hemoglobin, smoking status, initial wound area, and the wound location are presented in Table*2**.*

**Table 1:** Summary of the included studies

Study ID	Study design NCT	Sample Size	Follow-up duration	Definition of ulcers	Intervention applied			
					**Amniotic membrane products**	**Components**	**Processing method**	**Application**
**DiDomenico et al. 2016**	RCT NCT02399826	40	12 weeks	Ulcer with a size larger than 1 cm present for a minimum of 4wk duration, with no signs of infection	(Amnio Band, Musculoskeletal Transplant Foundation)	Amnion and chorion	Dehydrated	Graft
**DiDomenico et al. 2018**	RCT NCT02399826	80	12 weeks	Ulcer with a size larger than 1cm2 present for a minimum of 4wk duration, with no signs of infection	(Amnio Band Membrane, MTF)	Amnion and chorion	Dehydrated	Graft
**Lavery et al. 2014**	RCT N/A	97	12 weeks	Wound present between 4 and 52 weeks, wound located below the malleoli on plantar or dorsal surface of the foot and ulcer between 1 and 15 cm2	Grafix	Amnion/ chorion	Cryposervation	N/A
**Zelen et al. 2013**	RCT NCT01552499	25	12 weeks	Ulcer size > 1 and ulcer duration of ≥ 4 weeks; no clinical signs of infection	EpiFix	Amnion	Dehydrated	Graft
**Knowlton et al**	RCT NCT03547635	78	12 weeks	Ulcer size (i.e., area) is > 1 cm2 and < 12 cm2	AMNIOEXCEL	Amnion and chorion	Dehydrated	Graft
**Serena et al. 2019**	RCT N/A	76	16 weeks	Ulcer between 1 and 25 cm2	N/A	Amnion	Hypothermically	Graft
**Snyder et al. 2016**	RCT NCT02209051	29	6 weeks	The Wound that located superficially on the foot, distal to malleolus, Wagner grade 1 or 2, has a duration of at least 1 month with no clinical signs of infection or osteomyelitis, and between 1 cm2 and 25 cm2 in area	N/A	Amnion	Dehydrated	Graft
**Tettelbach et al. 2019**	RCT NCT01693133	110	12 weeks	Ulcer size ≥ 1 cm2 and < 25 cm2 and Ulcer duration of ≥ 4 weeks, unresponsive to standard wound care	N/A	Amnion and chorion	Dehydrated	Graft
**Thompson et al. 2019**	RCT N/A	13	12 weeks	Ulcer located on the plantar surface larger than 0.5 cm2	(Amnio Excel; Integra Lifesciences, Plainsboro, New Jersey)	Amnion	Dehydrated	Graft
**Zelen et al. 2014**	RCT NCT01921491	40	12 weeks	Ulcer size > 1 and < 25 cm2; ulcer duration of ≥ 4 weeks; no clinical signs of infection	EpiFix	Amnion and chorion	Dehydrated	Graft
**Zelen et al. 2016**	RCT NCT01921491	67	12 weeks	Ulcer size ≥ 1 and < 25 cm2.Ulcer duration of ≥ 4 weeks, unresponsive to standard wound care, no clinical signs of infection	EpiFix	Amnion and chorion	Dehydrated	Graft

*RCT* Randomized controlled trial, *MTF* Musculoskeletal trans-plant foundation, *N/A* Not available

**Table 2:** Baseline Characteristics of the included studies

Study ID	Study Arms	Sample	Age, mean (SD)	Gender (N)		BMI, mean (SD)	Glycated Hemoglobin, mean (SD)	Smoking Status (N)	Initial wound area (cm2), mean (SD)	Wound location			
				**Male**	**Females**					**Toe**	**Forefoot**	**Midfoot**	**Heel/ankle/ hindfoot**
**DiDomenico et al. 2016**	dHACA + SOC	20	59 (13)	11	9	37 (9.6)	7.5 (1.2)	4	2 (0.9)	4	7	8	1
	SOC alone	20	58 (9)	16	4	37 (11)	7.8 (1.5)	2	3.3 (4.35)	4	5	8	3
**DiDomenico et al. 2018**	dHACA + SOC	40	60.1 (11.7)	23	17	34 (9.3)	7.6 (1.47)	4	2.1 (1.46)	10	16	12	2
	SOC alone	40	61 (10.66)	31	9	34.5 (9.42)	7.9 (1.48)	3	3.1 (3.58)	3	14	12	5
**Lavery et al. 2014**	HVWM (Grafix®)	50	55⋅5 (11.5)	33	17	33⋅5 (7.7)	8 (1.6)	-	3⋅41 (3.23)	-	-	-	-
	Standard wound care	47	55.1 (12)	35	12	32.2 (7.9)	7.8 (1.5)	-	3.93 (3.22)	-	-	-	-
**Zelen et al. 2012**	Standard care	12	56.4 (14.7)	9	4	30.4 (5.7)	-	-	2.6 (1.9)	-	7	-	6
	EpiFix	13	61·7 (10.3)	7	5	35·4 (6.6)	-	-	3.4 (2.9)	-	7	-	5
**Knowlton et al**	AMNIOEXCEL (DAMA)	41	57.2 (11.32)	25	16	-	-	-	-	-	-	-	-
	Standard care	37	59.1 (11.14)	31	6	-	-	-	-	-	-	-	-
**Serena et al. 2019**	HSAM + SOC	38	59.2 (7.61)	30	8	-	-	-	3.12 (3.86)	-	-	-	-
	SOC alone	38	59.6 (10.72)	29	9	-	-	-	3.33 (4.62)	-	-	-	-
**Snyder et al. 2016**	AMNIOEXCEL (DAMA) + SOC	15	57.9 (12.49)	12	3	34.9 (5.9)	-	-	4.7 (5.43)	1	9	3	2
	SOC alone	14	58.6 (6.97)	13	1	35.1 (8.1)	-	-	6.9 (6.75)	0	6	5	3
**Tettelbach et al. 2019**	dHACM	54	57.4 (10.6)	40	14	35.8 (8.9)	7.8 (1.4)	22	3.2 (2.8)	4	27	8	8
	SOC alone	56	57.1 (10.5)	40	16	34.6 (8.5)	8.8 (1.8)	17	3.9 (3.8)	7	30	8	7
**Thompson et al. 2019**	Human amniotic allograft + skin substitute + Total contact case	7	58.5 (12.96)	6	1	**-**	9.63 (2.77)	-	1.54 (1.74)	-	-	-	-
	SOC + Total contact case	6	55.17 (18.32)	5	1	**-**	8.47 (2.44)	-	2.78 (3.04)	-	-	-	-
**Zelen et al. 2015**	Epifix	20	63⋅2 (13)	10	10	35 (7.5)	7.4 (1.5)	5	2.7 (2.4)	4	7	2	1
	SOC alone	20	62.2 (12.8)	9	11	35.8 (9.7)	8 (1.5)	5	3.3 (2.7)	5	5	3	3
**Zelen et al. 2016**	Epifix	32	63.3 (12.25)	19	13	33.9 (6.9)	7.5 (1.51)	9	2.6 (2.97)	9	9	8	6
	SOC alone	35	60.6 (11.55)	22	13	34.7 (9.35)	8.2 (1.78)	12	3.1 (3.17)	11	12	6	6

*dHACA* Dehydrated human amnion and chorion allograft, *SOC* Standard of care, *HVWM* Human viable wound matrix, *DAMA* Dehydrated amniotic membrane allograft, *HSAM* Hypothermically stored amniotic membrane

*dHACM* dehydrated human amnion/chorion membrane allograft, *SD* Standard deviation *N* Number, *BMI* Body mass index

### Outcomes

#### Complete wound healing

**Fig. 4:**
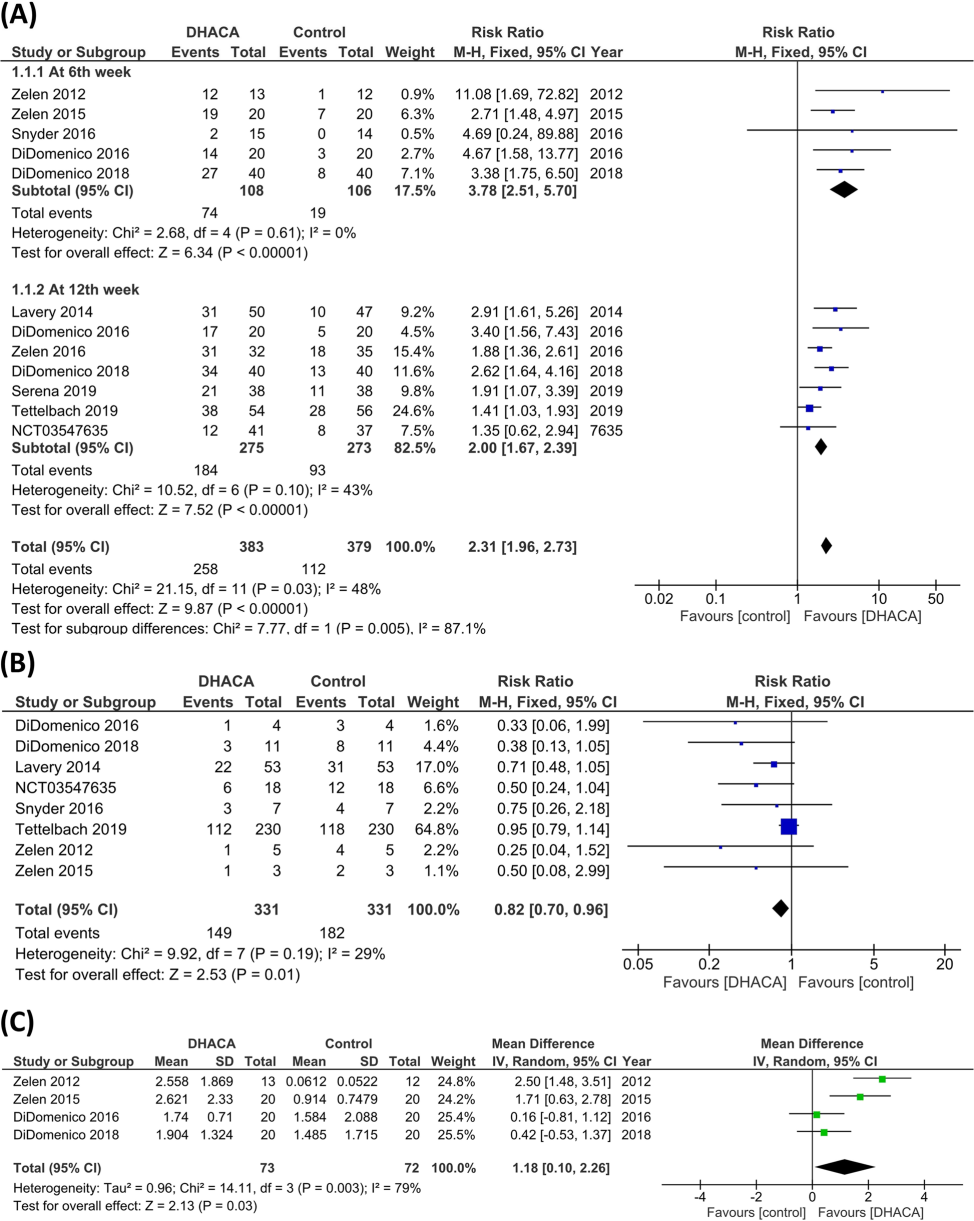
A comparison between (DHACA) + (SOC) group and the (SOC) alone group in terms of **(A)** complete wound healing. **(B)** risk of adverse events. **(C)** Mean difference Wound size reduction

The pooled results of the included studies showed a significant difference between DHACA plus SOC and the SOC alone, favoring the experimental group after the 6^th^ and 12^th^ weeks of follow-up (RR = 3.78; 95%CI: [2.51, 5.70], *P* < 0.00001) and (RR = 2.00; 95% CI: [1.67, 2.39], P < 0.00001) respectively. The pooled studies were homogenous in the 6^th^ week while heterogenous in the 12^th^ week (I^2^ = 0%, *P* = 0.61) and (I^2^ = 43%, *P* = 0.01) respectively. (Fig. 4a***).***

#### Adverse events

The analysis showed a significant difference between DHACA with SOC group and the SOC group favoring the experimental group (RR = 0.82, 95% CI: [0.70, 0.96], *P* = 0.01). The pooled studies' results were homogeneous (I^2^ = 29%, *P* = 0.19). (Fig. 4b)*.*

#### Wound size reduction

The pooled analysis of wound size reduction significantly favored DHACA with SOC over the SOC alone (MD = 1.18; 95% CI: [0.10, 2.26], *P* = 0.03). The pooled studies were heterogeneous and the heterogeneity could not be resolved (I^2^ = 79%, *P* = 0.003). (Fig. 4c)*.*

#### Time to heal

The analysis favored the DHACA group over the control group after the 1^st^ week of follow-up (RR = 5.74; 95%CI: [2.04, 16.18], *P* = 0.0009) as well as after the 6^th^ and 12^th^ weeks (RR = 3.00; 95%CI: [2.26, 3.98], *P* = 0.00001), (RR = 1.82; 95%CI: [1.46, 2.27], P = 0.00001) respectively. The results were significant in the three durations of follow-up with no inter-heterogeneity among the studies in the 1^st^, 6^th^, and 12^th^ weeks (I^2^ = 0%, *P* = 0.98), (I^2^ = 5%, *P* = 0.39), and (I^2^ = 17%, *P* = 0.31) respectively. (Fig. S.1)*.*

#### Kaplan–meier plot of time to heal

The pooled effect estimate of the included studies showed no significant difference between the two groups in the 4^th^ week (MD = -3.42; 95%CI: [-8.82, 1.97], *P* = 0.21), and the 6^th^ week (MD = -2.92; 95% CI: [-6.10, 0.26], *P* = 0.07). On the other hand, the analysis favored the experimental group in the 12^th^ week of follow-up (MD = -12.07; 95% CI: [-19.23, -4.91], *P* = 0.001). The results of pooled studies were heterogenous in the analyses of the 4^th^, 6^th^, and 12^th^ weeks (I^2^ = 92%, *P* < 0.00001), (I^2^ = 66%, *P* = 0.01), and (I^2^ = 71%, *P* = 0.004) respectively. (Fig. S.2)*.*

## Discussion

This systematic review and meta-analysis is based on ten published RCTs [12, 17, 18, 20–26] and one unpublished RCT (NCT03547635) that compared DHACA with SOC versus SOC alone in the treatment of DFUs. A total of 655 patients suffering from DFU were included in this systematic review. The meta-analysis findings showed that using DHACA with SOC is more effective and safer than the SOC alone for treating chronic DFUs. The pooled effect estimate of the 11 RCTs showed the superiority of the DHACA regarding complete wound healing in both the 6^th^ and 12^th^ weeks. The mean time for healing was not significantly different between the two groups at the 4^th^ and 6^th^ weeks, while a significant reduction in healing time was observed in the 12^th^ week favoring DHACA. Kaplan–Meier's plot of time to heal was significantly better in DHACA with SOC than SOC alone in the 1^st^, 6^th^, and 12^th^ weeks. Moreover, the current meta-analysis results revealed that DHACA can significantly reduce the wound size with a low risk of adverse events compared to SOC alone.

Similar to our findings, a previous meta-analysis [27] reported that the incomplete wound healing outcomes are less associated with DHACA plus SOC group than SOC only group at the 4^th^, 6^th^, and 12^th^ weeks with significant P values of < 0.0001, < 0.0001, and < 0.0001 respectively. This meta-analysis was conducted on seven studies with a total sample size of 347 patients and was limited by assessing a single outcome (wound healing). Contrary to the previous meta-analysis [27], our meta-analysis evaluated five outcomes: the percentage of complete wound healing, mean time to heal, Kaplan–Meier plot of time to heal, wound size reduction, and adverse events.

The human amniotic membrane is structured from three types of material: active cells, collagen fibers, extracellular matrix, and regenerative molecules. The amniotic membrane has been studied to investigate its effects on the wound healing process [28]. DiDomenico et al. 2016 [22] demonstrated that the mean and median time for wound healing is 12 weeks in the DHACA group, which was faster than most of the other cellular and/or tissue-based products (CTPs) reported in other RCTs [21, 25, 28–35]. In the multicenter trial Reyzelman et al. [31], 69.6% of the allograft has healed. While in Niezgoda et al. [34] 49% of small intestine submucosal CTP has healed. These findings reflect that DHACA might be promising and the most effective CTPs available.

Wound infections developed in DFU patients have 56 times the risk of requiring hospitalization and 155 times the risk of requiring amputation when compared to other wounds [36] . Once the patient’s foot or leg is amputated, an increase in the risk of repeated infections and ulcers arises [37]. To achieve wound healing, a 100% epithelialization must occur without drainage or need for dressing [23]. The main goal of DFU treatment is to enhance and facilitate complete wound healing; therefore, reducing the risk of complications such as infection, amputation, and delayed wound healing [27].

For our included studies, Zelen et al. [21] reported that complete wound healing occurred in 73%, 97%, and 51% of patients treated with bioengineered skin substitutes (BSS), dHACM, and SOC alone within 12 weeks, respectively. DiDomenico et al. 2016 [23] & DiDomenico et al. 2018 [22] showed that at the 12^th^ week, 85% of the DHACA-treated DFUs healed compared with 25% and 33% when treated with SOC alone, respectively. The mean time to heal ranged between 36 and 70 days in DiDomenico et al. 2016 [23] and between 37 and 67 days in DiDomenico et al. 2018 [22]. In addition, they concluded that the DHACA graft might have a sufficient clinical effect to be used in patients with more complex deep wounds that reach tendon and bone.

Lavery et al. [25] reported that the incidence of adverse events was 44% in Grafix group versus 66% in the SOC group, and the wound-related infections were fewer in the Grafix group (18%) than in the SOC group (36.2%). Similar to these findings, Zelen et al. 2013 [17] findings demonstrated that of patients who experienced DFU-related complications, 92% have healed with dehydrated human amniotic membrane allografts (EpiFix), while only 8% have healed with SOC alone. Zelen et al. 2015 [24] compared the median time to wound healing in DFPs using EpiFix, Apligraf, and SOC, they found that the healing time was significantly faster in EpiFix (13 days) compared to Apligraf (49 days) or standard care (49 days).

This meta-analysis is based on RCTs, which is considered a point of strength, the findings should be cautiously interpreted due to several concerns. The first concern is that dietary factors that could vary in other populations might have affected the generalizability of the studies' results. The second concern is about the possibility of patients' overlap in included studies. The third concern is the high heterogeneity in some outcomes that could not be resolved. Including English studies only could be considered a limitation of the current review. In addition, a possible concern can arise in studies by Zelen et al. 2013 [17], Zelen et al. 2015 [24], and Zelen et al. 2016 [21], also in DiDomenico et al. 2016 [23] and DiDomenico et al. 2018 [22] since these studies were conducted by the same authors. Therefore, future studies from different countries/populations are necessary to explore the effect of DHACA in treating DFUs in other populations.

## Conclusion

The current review results support that DHACA with SOC has better efficacy than SOC alone in enhancing wound healing, reducing the mean time to wound healing, and diminishing the risk of adverse events. All these findings are in turn beneficial for treating DFUs patients.

## Supplementary Information

**Additional file 1:** **Figure S.1.** Forest plot of risk ratio (RR) in time to heal after 4th, 6th, 12th follow-up, between (DHACA)+(SOC) group and the (SOC) alone group. **Figure S.2.** Forest plot of mean difference (MD) in Kaplan–Meier plot of time to heal within 1st, 6^th,^ and 12^th^ weeks follow-up, between (DHACA)+(SOC) group and the (SOC) alone group.

## Data Availability

All data are available upon reasonable request from the corresponding author.
