# Supplementary material for: Human amniotic membrane products for patients with diabetic foot ulcers. do they help? a systematic review and meta-analysis
Source: J Foot Ankle Res. 2022 Sep 14;15:71. doi: 10.1186/s13047-022-00575-y (PMC9472416; doi:10.1186/s13047-022-00575-y)

Human amniotic membrane products for patients with diabetic foot ulcers. Do they help? A Systematic Review and Meta-analysis.

**Authors’ Names**

**Authors’ Names**

Yasmine Adel Mohammed ^*1,6^, Hossam Khaled Farouk ^2,6^, Mohamed Ibrahim Gbreel ^3,6^, Abdelrahman Mahmoud Ali ^4,6^, Ali Ashraf Salah ^4,6^, Anas Zakarya Nourelden ^5,6^, Mohamed Mahmoud Abd-El Gawad ^2,6^

**Authors’ affiliations**

**^1^** Faculty of Medicine, Assiut University, Assiut, Egypt.

**^2^** Faculty of Medicine, Al-Fayoum University, Fayoum, Egypt.

**^3^** Faculty of Medicine, October 6 University, Giza, Egypt.

**^4^** Faculty of Medicine, Minia University, Minia. Egypt.

^5^Faculty of Medicine, Al-Azhar University, Cairo, Egypt.

**^6^** International Medical Research Association (IMedRA), Egypt.

***Correspondence to:** Yasmine Adel Mohammed, Faculty of Medicine, Assiut University, Assiut, Egypt. **Postal code:** 71631, **Address:** Assiut governorate, Egypt, **Tel:** +0201126057816, **Email:** [yasminea126@gmail.com](mailto:yasminea126@gmail.com)

**(Supplementary 1)**


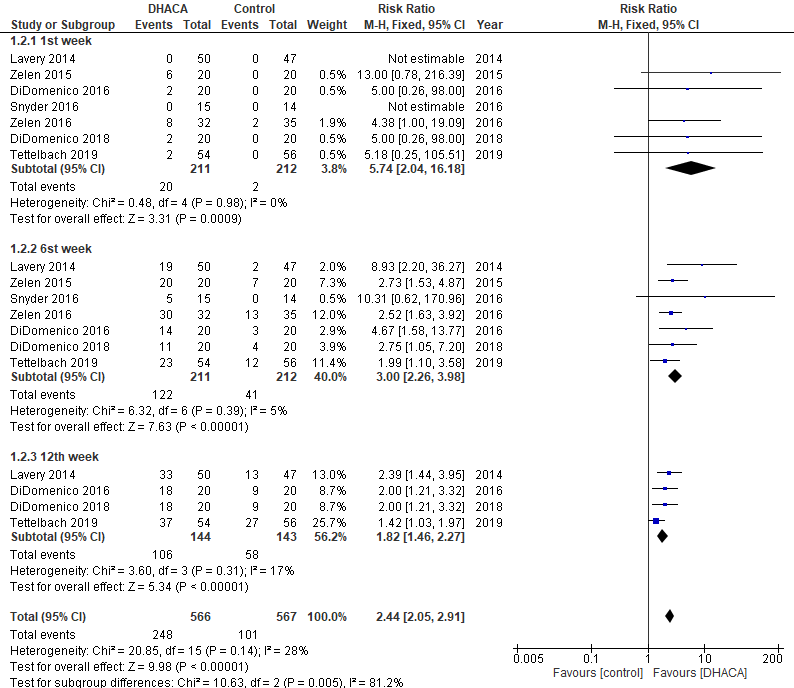
**Figure S.1.** Forest plot of risk ratio (RR) in time to heal after 4^th^, 6^th^, 12^th^ follow-up durations

**Figure S.2.** Forest plot of mean difference (MD) in Kaplan–Meier plot of time to heal within 1^st^, 6^th^ and 12^th^ weeks


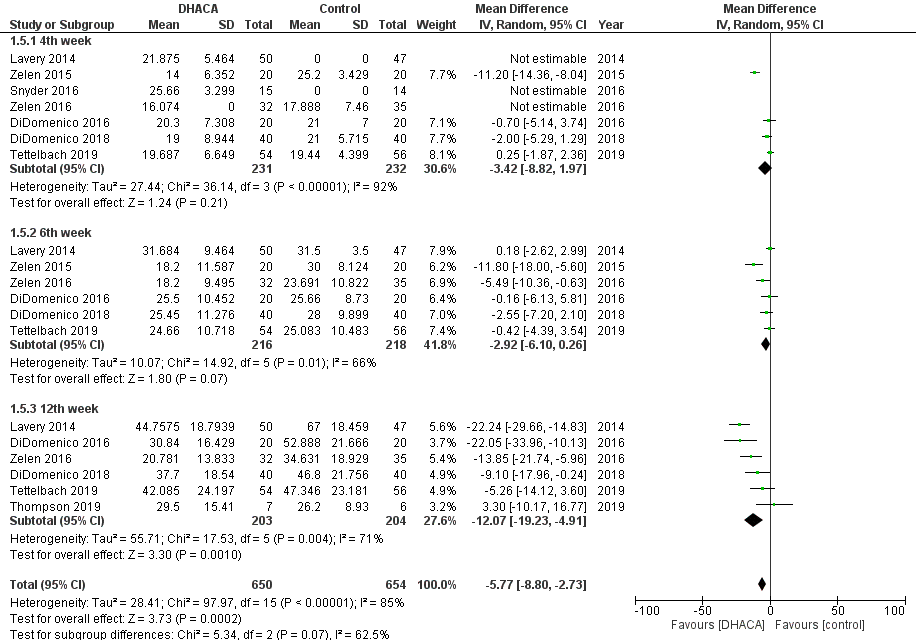

Supplement: Supplementary file 1 — Additional file 1: Figure S.1. Forest plot of risk ratio (RR) in time to heal after 4th, 6th, 12th follow-up, between (DHACA)+(SOC) group and the (SOC) alone group. Figure S.2. Forest plot of mean difference (MD) in Kaplan–Meier plot of time to heal within 1st, 6th, and 12th weeks follow-up, between (DHACA)+(SOC) group and the (SOC) alone group. [file 13047_2022_575_MOESM1_ESM.docx]
